# Single-Prolonged Stress Induces Endoplasmic Reticulum - Dependent Apoptosis in the Hippocampus in a Rat Model of Post-Traumatic Stress Disorder

**DOI:** 10.1371/journal.pone.0069340

**Published:** 2013-07-19

**Authors:** Fang Han, Shengnan Yan, YuXiu Shi

**Affiliations:** PTSD Laboratory, Department of Histology and Embryology, Institute of Pathology and Pathophysiology, China Medical University, Shenyang, China; Emory University, United States of America

## Abstract

**Background:**

Our previous research indicated that apoptosis induced atrophy in the hippocampus of post-traumatic stress disorder (PTSD) rats. Endoplasmic reticulum (ER) stress-induced apoptosis has been implicated in the development of several disorder diseases. The aim of this study was to investigate whether endoplasmic reticulum-related pathway is involved in single-prolonged stress (SPS) induces apoptosis in the hippocampus of PTSD rats by examining the expression levels of three important indicators in the ER-related apoptotic pathway: Glucose-regulated protein (GRP) 78, caspase-12 and Ca^2+^/CaM/CaMkinaseIIα (CaMkIIα).

**Methods:**

Wistar rats were sacrificed at 1, 4 and 7 days after SPS. SPS is a reliable animal model of PTSD. The apoptotic cells in the hippocampus were assessed by TUNEL method and transmission electron microscopy (TEM). Free intracellular Ca^2+^ concentration was measured. GRP78 expression was examined by immunohistochemistry, western blotting and RT-PCR. mRNA of caspase-12 and CaM/CaMkIIα were determined by RT-PCR.

**Results:**

Our results showed that apoptotic cells were increased in the SPS rats. TEM analysis revealed characteristic morphological changes of apoptosis in these cells. We observed that GRP78 was significantly up-regulated during early PTSD, and then recovered at 7 days after SPS. By RT-PCR, we observed that the change in caspase-12 expression level was similar to that in GRP78. Moreover, the free intracellular Ca^2+^ concentration was significantly higher at 1 day after SPS and decreased in 7 days. CaM expression increased significantly, while CaMKIIα expression decreased significantly in the hippocampus at 1 day after SPS.

**Conclusion:**

SPS induced change in the expression levels of GRP78, caspase-12 and Ca^2+^/CaM/CaMkIIα in the hippocampus of PTSD rats indicated that the endoplasmic reticulum pathway may be involved in PTSD-induced apoptosis.

## Introduction

Post-traumatic stress disorder (PTSD) is an anxiety disorder that may develop following exposure to a death threat or serious injury. This may cause affected individuals to continuously re-experience the traumatic event [1], [2] and react with intense fear, helplessness or horror for years. Impaired hippocampal function is one of the various causes of PTSD [3]. Many studies have found that the hippocampal volume is significantly smaller in PTSD patients [4], [5], [6], [7]. In the past several years, our research team examined apoptosis in the smaller hippocampus of rats modeled with PTSD by using single prolonged stress (SPS) [8], [9], [10], which is a reliable animal model of PTSD based on the time-dependent dysregulation of the hypothalamic–pituitary–adrenal (HPA) axis [11], [12].

Apoptosis is a genetically controlled and complex process central to development, homeostasis and disease. It is activated in response to environmental signals or by intrinsic factors, and designed to kill errant cells in an orderly and clean manner [13], [14]. According to various apoptotic stimuli, apoptosis can be induced by two major pathways: the intrinsic pathway (mitochondria-dependent pathway) and the extrinsic pathway (death receptor-dependent pathway).

Another type of intrinsic pathway begins with the activation of a defensive response by the endoplasmic reticulum (ER). ER is an essential intracellular organelle which is responsible for the synthesis and maturation of cell surface and secretion proteins, and maintenance of Ca^2+^ homeostasis. Disruption of these physiological functions leads to accumulation of unfolded proteins and induces the unfolded protein response (UPR) [15], [16]. If the stress on the ER is excessive or prolonged, UPR initiates the apoptotic cell-death cascade [17], [18]. A major UPR upregulated target protein is the glucose regulated protein 78 kDa (GRP78). GRP78 is involved in many cellular processes, including translocation of newly synthesized polypeptides across the ER membrane, folding and assembly of newly synthesized proteins, maintenance of these proteins in a state competent for subsequent folding and oligomerization, and regulation of Ca^2+^ homeostasis [19], [20]. In addition to its chaperone function, GRP78 is a key regulator of ER stress transducers. GRP78 binds PERK, IRE1 and ATF6 and inhibits their activation in non-stressed cells [21]. Upon ER stress and accumulation of unfolded proteins in the ER, these molecules are released from GRP78 and become activated to control transcription, translation, and apoptosis. Recent studies have revealed that GRP78 is anti-apoptotic and plays critical cytoprotective roles in neurodegenerative diseases [10], [22].

Caspases, a family of cysteine-dependent aspartate-specific proteases, are critical mediators of apoptosis. Fourteen members of the caspase family have been identified and shown to be widely expressed in a variety of tissues and cell types. Caspases normally exist in cells as proenzymes, which may be activated through recruitment into activating complexes or direct proteolytic cleavage by another caspase [23]. Among the caspase family members, caspase-12 is a key signal involved in ER stress-induced apoptosis. Caspase-12 is localized on the cytoplasmic side of the ER. It is also specifically activated in cells treated with ER stress-agents, but not in cells treated with death cytokines or intrinsic apoptotic stimuli. It has been shown that cells from caspase-12 deficient mice are resistant to apoptosis triggered by the known ER stress agents [17].

Calcium (Ca^2+^), one of the key elements of apoptotic signalling pathways [24], is a universal signaling molecule regulating many aspects of cellular function. Dysregulation of intracellular Ca^2+^ homeostasis results in the activation of apoptotic pathways [24]. Calmodulin (CaM) is the major Ca^2+^ sensor in nonmuscle cells [25] and signaling involving calmodulin has been implicated in apoptosis [26]. Different signaling mechanisms downstream of CaM is involved in various types of apoptotic responses, including pathways involving calcineurin, DAP kinase and calmodulin kinases. Calmodulin-dependent Kinase IIα (CaMKIIα) has been found to be both pro-apoptotic [27] and anti-apoptotic in different studies. When the Ca^2+^ concentration increases, Ca^2+^, calmodulin (CaM) and CaM kinase IIα (CaMKIIα) combine together to form the Ca^2+^ -CaM-CaMKIIα signaling pathway, which is important in the plasticity of the central nervous system, learning and memory, behavior and other types of cognitive activities.

In the present study, we mainly focused on three important indicators in the endoplasmic reticulum pathway: GRP 78, Caspase-12 and Ca^2+^/CaM/CaMKIIα, to examine the SPS-induced apoptosis in the hippocampus involved in endoplasmic reticulum pathway.

## Materials and Methods

### Animal Model Preparation and Grouping

A total of 100 male Wistar rats (8–10 weeks, 150–180 g) were randomly divided into four groups: a control group, SPS groups examined on day 1 (1-day), day 4 (4-day) and day 7 (7-day). The control rats remained in their home cages with no handling for 7 days and were sacrificed at the same time as the SPS groups. The SPS rats underwent the SPS procedure on the first day. The SPS protocol [8] consisted of a 2-h immobilization (compression with plastic bags), 20-min forced swim (25°C), 15-min rest, followed by ether anesthesia (until loss of consciousness). Following SPS, the rats were fed routinely. The study was approved by the ethics committee of China Medical University. Experiments were carried out in accordance with the Guidelines laid down by the NIH in the US regarding the care and use of animals for experimental procedures.

### Perfusion-based Sections

Rats of both the normal control group and SPS groups were prepared by left ventricle perfusion with 4% paraformaldehyde in phosphate buffer (PB) and fixation, and post-fixed in the same fixative at 4°C for 6–10 h. They were then immersed in a 20% sucrose solution in 0.01 M phosphate buffer (PB, pH 7.4) at 4°C. Samples were snap-frozen in liquid nitrogen and sectioned. Coronal sections (12-µm) were prepared for the morphological studies.

### Terminal Transferase dUTP Nick-end Labeling (TUNEL)

Dewaxed sections of 4 rats of each group were washed three times (5 min each) in 0.01 M PBS, and permeabilized in proteinase K for 10 min. Endogenous peroxidase was deactivated by 0.3% hydrogen peroxide. These sections were washed three times again. Then they were incubated with TDT at 37°C for 1 h, and incubated with antibody at 37°C for 1 h. These sections were stained by 3,30-diaminobenzidine (DAB), and after hematoxylin post-staining, were mounted and observed under light microscope.

Five slides were randomly selected from each group, and in each slide, five visual fields (X40) in the hippocampus were randomly selected. The number of TUNEL-positive cells was counted with about 500 cells counted per slide. The TUNEL-positive cells rate was calculated to equal (the number of TUNEL-positive cells/total cells) X100%.

### Assessing the Morphological Changes in the Hippocampus Using Transmission Electron Microscopy (TEM)

Under anesthesia, 4 rats per group were perfused with 4% paraformaldehyde and 2.5% glutaraldehyde in phosphate buffer (PB). The brains were removed and immediately immersed in 2.5% glutaraldehyde in 0.1 M PB, pH 7.4 on ice, with shaking for 6 h. After sufficient washing with 0.1 M PB, the brains were cut into blocks measuring about 1 mm wide, 5 mm long, and 1 mm thick with the hippocampus at the center of the blocks. The blocks were post-fixed in 1% osmium tetroxide for 2 h at 4°C. They were rinsed in distilled water for several times, dehydrated in graded series (20–100%) of ethanol and then in propylene oxide, infiltrated with Epon 812, and finally polymerized in pure Epon 812 for 48 h at 65°C. After polymerization, selected areas of the CA3 region including the pyramidal layer of the hippocampus were identified, trimmed, and mounted on blank plastic blocks. Ultra-thin sections were cut on an ultra microtome using diamond knives, collected on copper grids, and stained with 4% uranyl acetate and Reynolds lead citrate. A minimum of 10 sections from each hippocampus were observed under a transmission electron microscope (JEM-1200EX; JEOL, Tokyo, Japan).

### Immunohistochemistry for GRP78

Sixteen rats of control group and SPS groups (4 rats per group) were used to observe GRP78 immunoreactivity. Briefly, 5 sections per brain were selected for incubating with 0.3% hydrogen peroxide and methanol for 10 minutes and later boiled in 10 mM citrate buffer (pH 6.0) in a 1000 W microwave oven for 10 minutes. Tissue sections were then incubated with the primary antibodies overnight at 4°C. The primary antibody used was rabbit polyclonal antibody to GRP78 (1∶200) from Santa Cruz Biotechnology (Santa Cruz, CA, USA). Then the sections were incubated with two-step immunohistochemistry detection reagent (PV6001 and PV6002; Zhongshan Goldenbridge, Beijing, China) at 37°C for 30 minutes. A brown color appeared in the slices after 3,30-diaminobenzidine colorization. As a negative control, some sections were incubated without primary antibody and processed as described above. No distinct staining was observed.

### Western Blotting for GRP 78 and Caspase-12

Sixteen rats of control group and SPS groups (4 rats per group) were decapitated, and the brains were removed and immediately placed in an ice-cold dish. Then the hippocampus was dissected according to the atlas (Paxinos and Watson, 1998) by use of a stereomicroscope, quick frozen in liquid nitrogen and stored at −80°C. For GRP78, the hippocampus of normal control rats and SPS rats were homogenized with a sample buffer containing 200 mM TBS, pH 7.5, 4% SDS, 20% glycerol, 10% 2-mercaptoethanol and denatured by boiling for 3 min. For caspase-12, method of sub cellular fractionation was described on the next paragraph. Samples (50 lg/lane) were loaded on a 7.5% SDS–polyacrylamide gel, and electroblotted onto a PVDF membrane (Millipore Corp., MA, USA) by a semi-dry blotting apparatus (Bio-Rad Laboratories, Inc., Hercules, CZ). The blotted membrane was then blocked with 1.5% skim milk containing 0.05% Tween-20 in TBS (TBST) at 4°C overnight, and then incubated with 1∶500 rabbit polyclonal antibody against GRP 78 (Santa Cruz, CA, USA) and 1∶1000 rabbit polychonal antibody against casapse 12 (Santa Cruz, CA, USA) at 4°C for 24 h. Blots were washed three times with TBST, and then incubated with a second antibody (anti-rabbit IgG HRP from Santa Cruz; 1∶2000) for 2 h at room temperature. After incubation, blots were washed three times with TBST before visualization by enhanced chemiluminescence (ECL; Amersham Pharmacia Biotech, British). To confirm equal protein loading, the same blots were incubated with antibodies specific for β-actin (Abcam, British; 1∶1000). Immunoreactivity for β-actin was detected with the ECL. The optical density (OD) of GRP78, caspase 12 and β-actin was analyzed on the Gel Image Analysis System (Tanon 2500R, Shanghai, PR China). We repeated the experiment 3 times and had similar results.

### Sub Cellular Fractionation

The hippocampi were fractionated to obtain the cytoplasm, mitochondria and endoplasmic reticulum-containing (microsomal) fraction, according to previous methods [28]. Briefly, samples were homogenized in 1×M-SHE buffer (210 mM Mannitol, 70 mM Sucrose, 10 mM HEPES-KOH pH 7.4, 1 mM EDTA, 1 mM EGTA and protease inhibitor cocktail) and then centrifuged twice at 1200×*g* for 10 min. The post-nuclear supernatant was then centrifuged twice at 10,000×*g* for 15 min and the resulting mitochondrial pellet was resuspended in a sucrose buffer (395 mM sucrose, 0.1 mM EGTA, 10 mM HEPES-KOH pH 7.4) and purified through a percoll bilayer in gradient buffer (1.28 M sucrose, 0.4 mM EGTA, 40 mM HEPES-KOH, pH 7.4) by centrifugation at 41,000×*g* for 30 min. The crude cytosolic fraction was then centrifuged at 100,000×*g* for 1 h to separate the microsomal and cytosolic fractions.

### Determination of Free Calcium Content in the Hippocampal Cells

Three rats from each group were rapidly decapitated, and the brains were removed. The hippocampus was then dissected out according to the atlas of rat neuroanatomy using a stereomicroscope and minced. The minced tissue was made to the cell suspension according to previous method [29]. The hippocampal cell suspension was incubated with 1 mol/l Fura-2/AM (Beyotime, China) for 35 min, and then detected with a spectrofluorometer (F-4500FL, Hitachi High-technologies, Japan). The basal emission was measured by stimulating the cells with 340/380 nm light and recording the emitted fluorescence intensity at 510 nm. Approximately 3.5×10^6^ cells per sample was monitored. The intensity of fluorescence was calculated automatically. The *R*max and *R*min values were determined by the addition of Triton X-100 (0.1%) and EGTA (pH 8.7, EGTA final concentration was 5 mM), respectively. Calculation of Ca^2+^ was made with the following equation: Ca^2+^ = Kd(R−Rmin)/(Rmax −R) Fmin/Fmax, where Kd is the dissociation constant of Fura-2 for Ca^2+^ and is assumed to be 224 nM at 37°C. R is the ratio of corrected fluorescence at 340 and 380 nm. *R*max is the ratio obtained after 0.1% Triton X-100 treatment. *R*min is the ratio obtained after EGTA treatment.

### Reverse Transcription-polymerase Chain Reaction (RT-PCR) to Detect GRP 78, Caspase-12 and CaM/CaMK II

The hippocampal total mRNA from sixteen rats (4 rats per group) was extracted using the TRIzol kit according to the manufacturer’s instructions. The forward and reverse sequences of the primers (synthesized by Shenggong Biotech Co., Shanghai, China) were according to the serial numbers from GenBank and are listed in Table 1. Tissue samples were homogenized, and total RNA was extracted. PCR was performed as described previously. The cycling reaction for GRP 78 was as follows: 4 min polymerase activation at 95°C and amplification for 40 cycles of [95°C (30 s), 60°C (30 s), and 72°C (1 min)]. For CaM, the PCR profile used was 94°C for 4 min, and amplification for 32 cycles of [94°C (30 s), 58°C (30 s) and 72°C (40 s)]. For CaMKIIα, the reaction was started at 95°C for 2 min, followed by amplification for 33 cycles of [95°C (30 s), 55°C (30 s) and 72°C (40 s) and a final 5-min extension at 95°C. For Caspase 12, the PCR profile used was two cycles of 95°C, 65°C and 72°C; then two cycles of 95°C, 62.5°C and 72°C; β-actin mRNA used as an internal control. The products were observed following electrophoresis on a 1.2% agarose gel and the density of each band was analyzed on the Gel Image Analysis System. The levels of GRP78, CaM, CaMKIIα and Caspase-12 mRNA were determined by calculating the density ratios of GRP78mRNA/β -actin mRNA, CaM mRNA/β -actin mRNA, CaMKIIα mRNA/β-actin mRNA and Caspase-12 mRNA/β -actin mRNA. We repeated the experiment 3 times and had similar results.The following primers for RT- PCR were used (Table 1).

**Table 1:** All primers for RT-PCR.

Name	Primer	Product Size (bp)
GRP78	Sense 5′-CCAAGAGAGGGTTCTTGAATCTCG -3′antisense 5′-ATGGGCCAGCCTGGATATACAACA -3′	181 bp
CaM	Sense 5′-GGCATCCTGCTTTAGCCTGAG-3′antisense 5′-ACATGCTATCCCTCTCGTGTGAC-3′	328 bp
CaMKIIα	sense 5′-CATCCTCACCACTATGCTG-3′antisense 5′-ATCGATGAAAGTCCAGGCCG-3	284 bp
Capase-12	sense 5′-GCACATTCCTGGTCTTTATGTCCC-3′antisense 5′-GCCACTGCTGATACAGATGAGGAA -3′	312 bp
β-actin	sense 5′-GTCACCCACACTGTGCCCATCT-3′antisense5′-ACAGAGTACTTGCGCTCAGGAG-3′	542 bp

All primers were designed using DNAstar Primer Select program (Lasergene, Madison, WI, USA) and synthesized by Shanghai Sangong (Shanghai, China).

### Statistics

The results were expressed as mean ± S.D. The differences between normal control group and SPS groups were analyzed by one-way analysis of variance (ANOVA) followed by Tukey’s post-hoc test using SPSS 13.0 software. A level of P<0.05 was considered to be statistically significant.

## Results

### Apoptotic Cells in the Hippocampus was Detected by TUNEL Method

**Figure 1:**
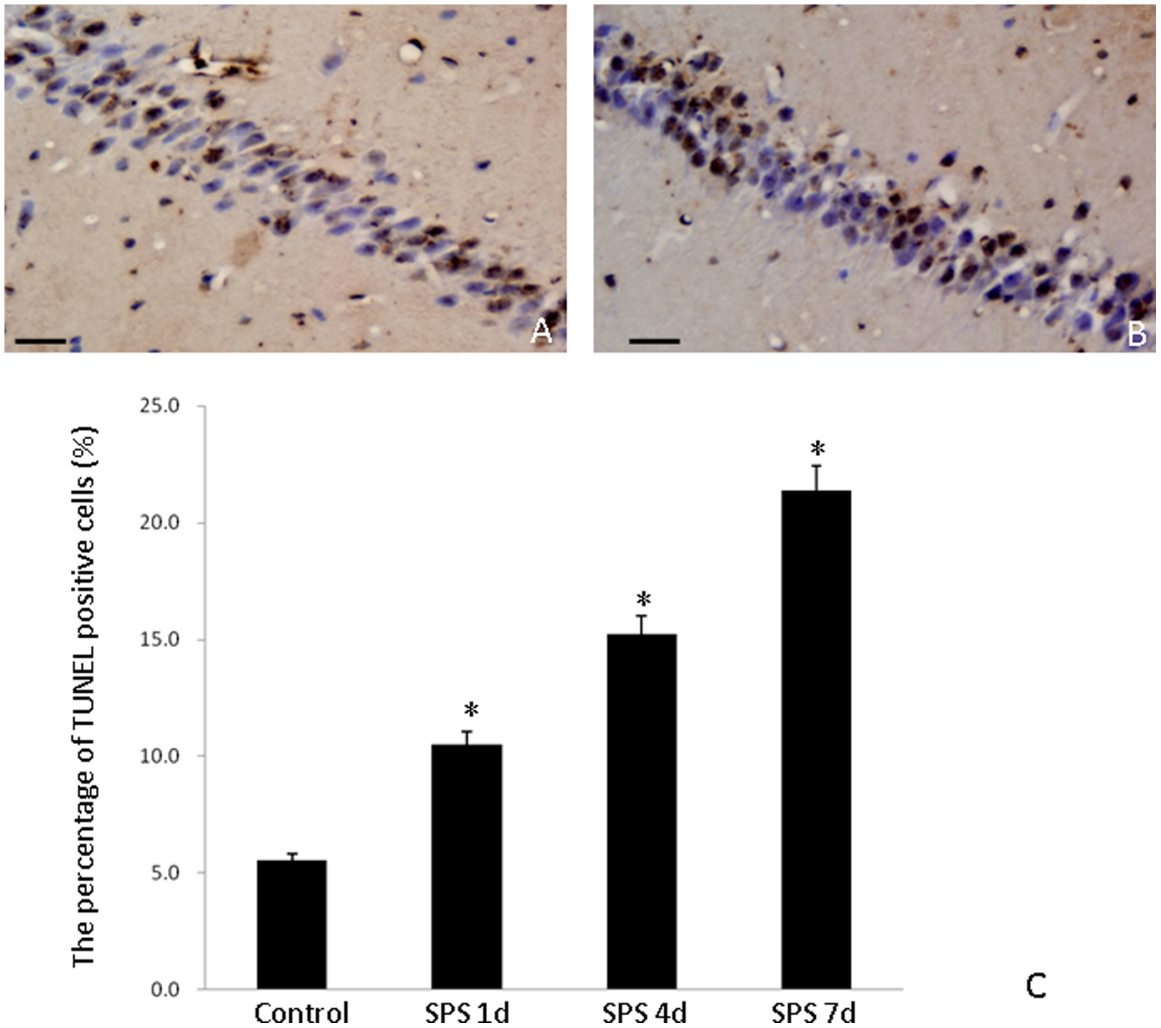
Apoptotic cells in the hippocampus was detected by TUNEL method. Morphological changes in apoptotic cells in the hippocampus of single prolonged stress (SPS) rats. The TUNEL assay was used to detect apoptotic cells in the hippocampus. (A) Control group; (B) 7 days after SPS; (C) quantification of TUNEL-positive cells (mean ± SD), *P<0.05 vs. the control group. Scale bar 520 µm.

The TUNEL-positive cells were rarely found in the hippocampus of the control group (Fig. 1A). In contrast, the total number of TUNEL-positive cells was consistently increased in the SPS rats (Fig. 1B, 1C).

### TEM Analysis of the Morphological Changes in Cells in the Hippocampus of the SPS Rats

**Figure 2:**
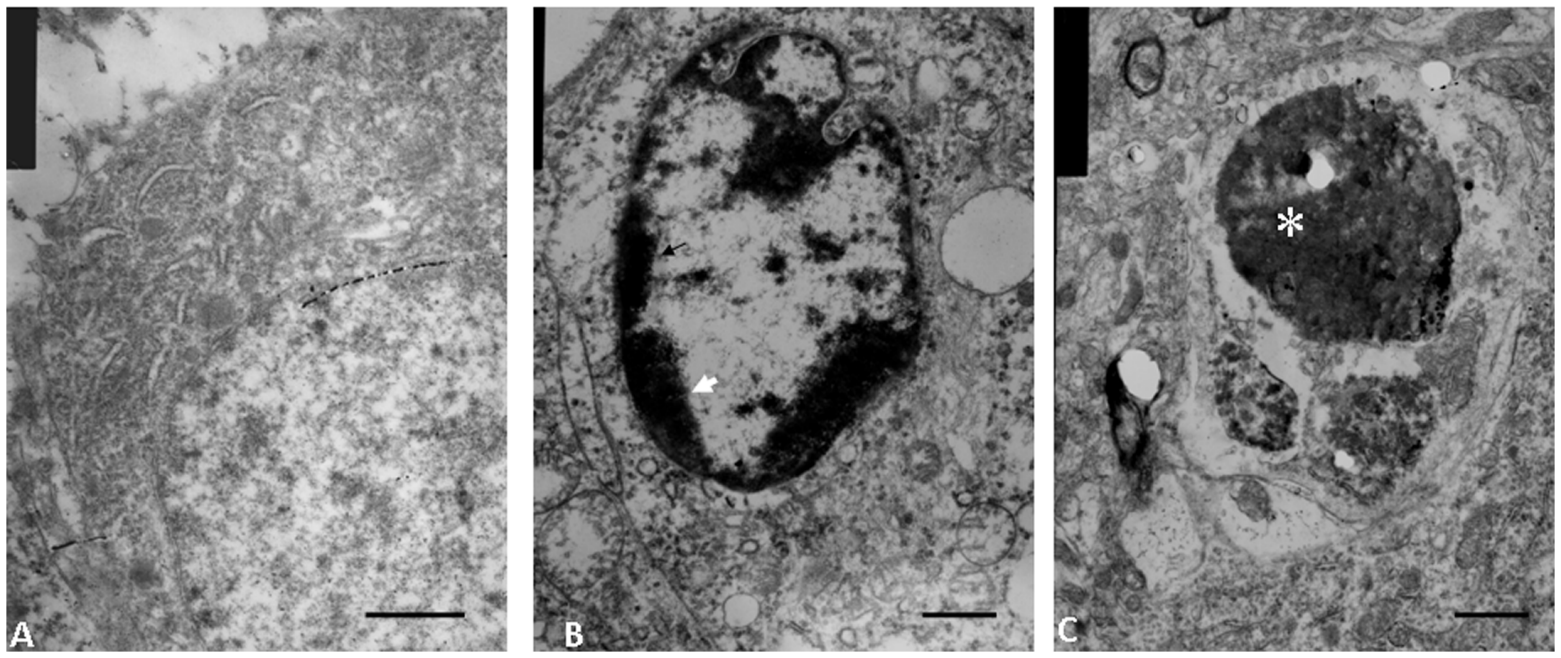
TEM images of the hippocampus in the SPS rats. TEM images of the hippocampus. A: Normal control hippocampal neuron, displaying abundant organelles such as mitochondria, endoplasmic reticulum, and ribosomes. The nucleus is large and round, density of chromatin is uniform, and nucleolus is clear. B: Chromatin margination, in the form of crescent formation in nucleus (arrow), is shown. C: The cells showed some typical structural changes indicative of apoptosis, such as chromatin dissolution and, nuclear envelope breakdown (star). Scale bar = 200 nm (A). Scale bar = 300 nm (B and C).

As shown in Fig. 2, the hippocampal cells in the control rats exhibited normal morphology (Fig. 2A). Some cells in the hippocampus of SPS rats (Figs. 2B, 2C) exhibited changes characteristic of apoptosis, including chromatin condensation, appearance of chromatin crescents (shown with arrow), nucleus fragmentation (shown with star) and nucleolus disappearance.

### Immunohistochemical Detection of GRP78

**Figure 3:**
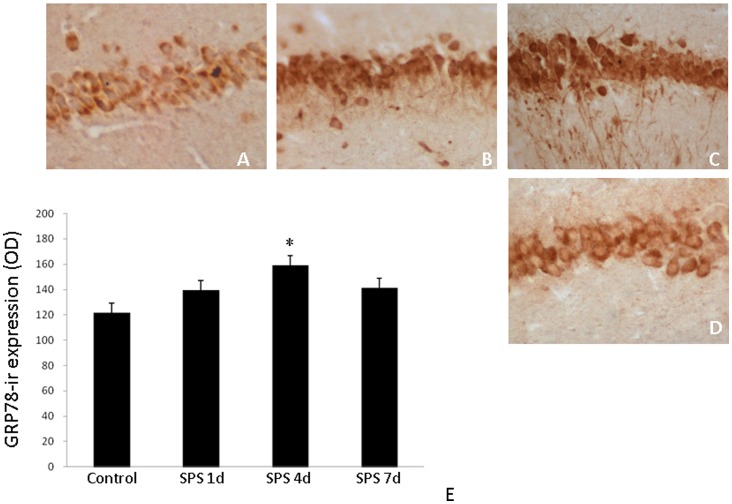
Immunohistochemical observation of GRP78. GRP78-immunoreactivity (ir) in the hippocampus of single prolonged stress (SPS) rats in different groups (A–D, magnificationx400). A: control group; B: SPS 1d; C: SPS 4d; D: SPS 7d; E: the quantity of GRP-ir expression (OD). *P<0.05 vs. the control group.

GRP78 (ir) was distributed mainly in the cytoplasm of the hippocampal cells in the control group (Fig. 3A). At 1 day after SPS, increased expression of GRP78-ir was observed (Fig. 3B, 3E), and then peaked at 4 days after SPS (Fig. 3C, 3E). In contrast, decreased expression of GRP78 was observed at 7 days after SPS (Fig. 3D, 3E).

### Western Blot Analysis of GRP78

Western blots to GRP 78 showed a single band at 78 KD, and the band density mean value of the control group was set as 100%. Data were expressed as normalized optical density. β -actin protein level was used as the internal control at each time point.

**Figure 4:**
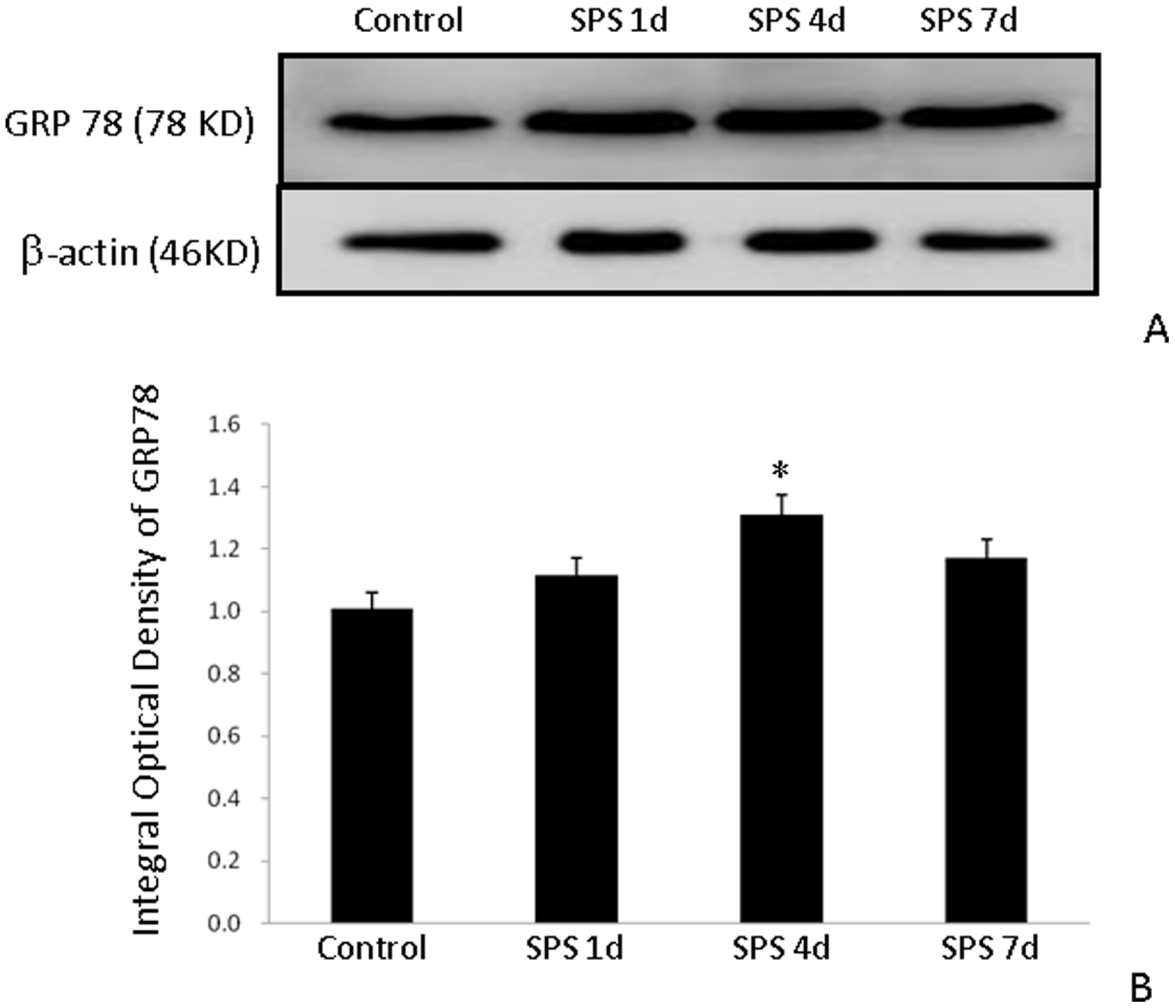
Western blot of GRP78 in the hippocampus of SPS rats. GRP78 protein expression (A) and results from its quantitative analysis based on western blot results (B). GRP78 expression was analyzed in the hippocampus of control rats and of rats subjected to single prolonged stress (SPS). An increase in GRP78 protein expression was observed in SPS rats. *P<0.05 vs. the control group.

In the SPS group, the expression of GRP78 protein in the hippocampus increased at 1 day after SPS and peaked at 4 day after SPS. The extent of decrease in the GRP78 protein level at 7 days after SPS group was significantly greater than that at 4 days after SPS (Fig. 4).

### GRP78 mRNA Expression

**Figure 5:**
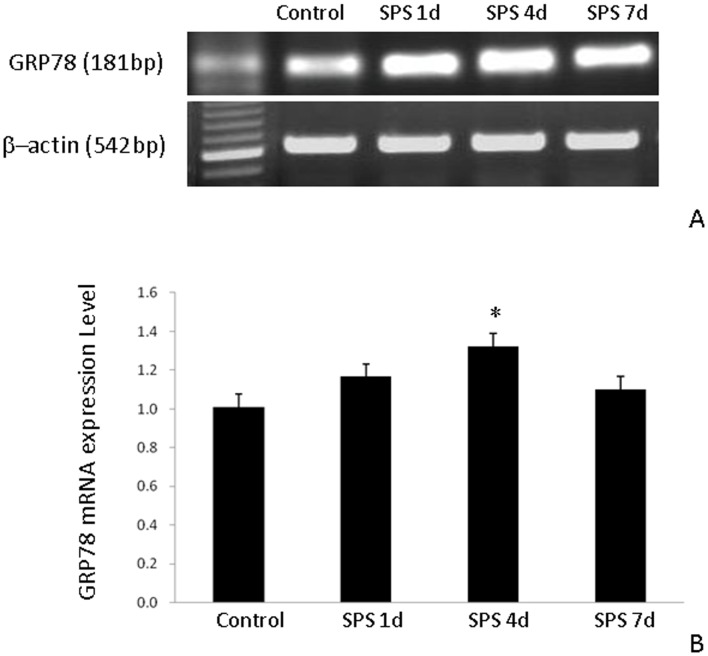
RT-PCR of GRP78 in the hippocampus of the SPS rats. GRP78 mRNA expression (A) and results from its quantitative analysis (B). GRP78 mRNA expression in the hippocampus of rats subjected to SPS was higher than that in the hippocampus of control rats. *P<0.05 vs. the control group.

The mRNA expression level of GRP78 in the hippocampus of the SPS group was higher than that of the control group. At 4 day after SPS, mRNA expression of GRP78 peaked and then decreased at 7 days after SPS (Fig. 5A, 5B). The mRNA expression data were consistent with western blot data.

### Western Blot Analysis of Casapse-12

**Figure 6:**
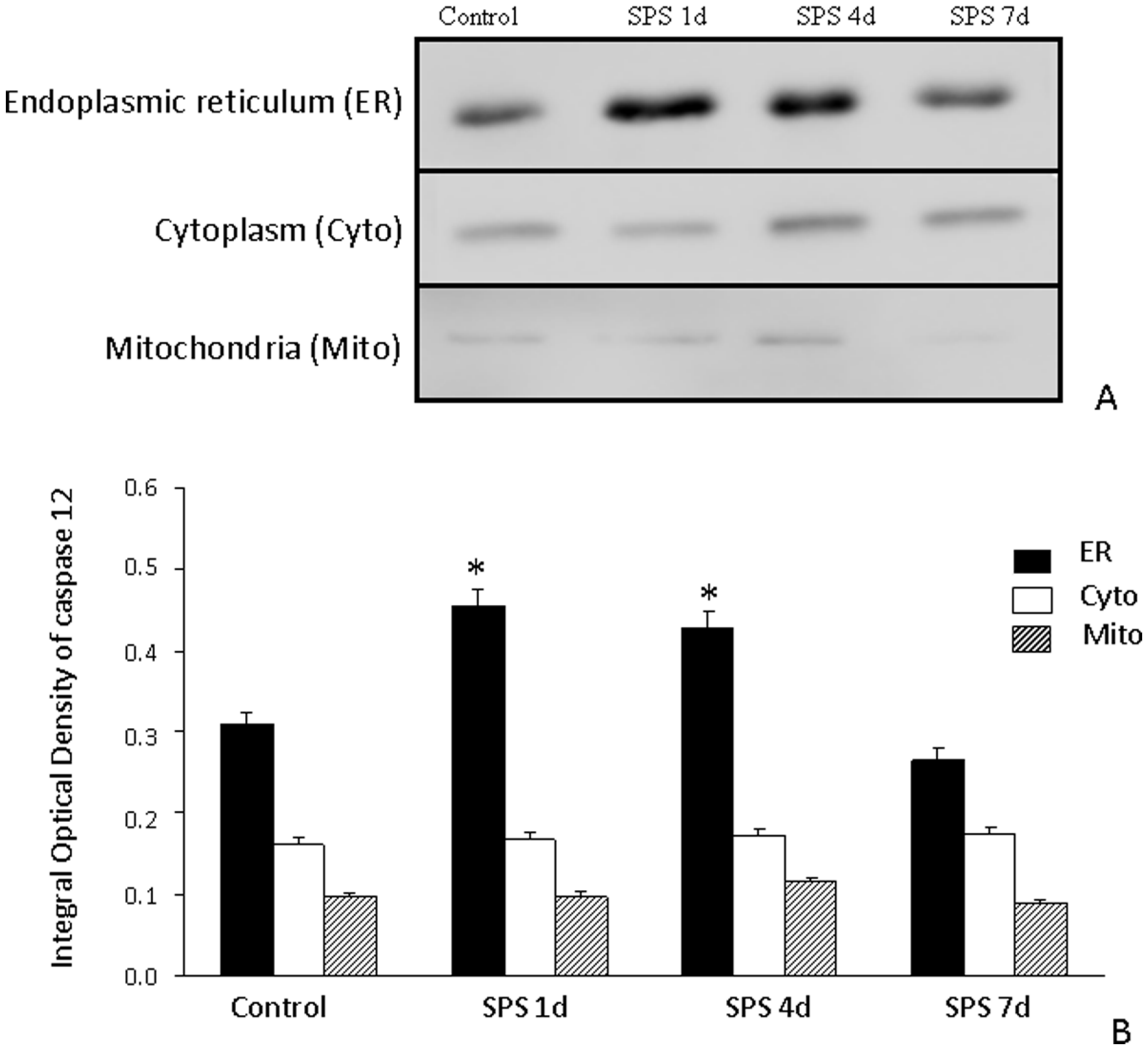
Western blot of caspase-12 in the hippocampus of SPS rats. Caspase-12 protein expression in the endoplasmic recutilum (ER), cytoplasm (Cyto) and mitochondira (Mito) fractions of the hippocampal cells (A) and results from its quantitative analysis based on western blot results (B). An increase in caspase-12 protein expression in the ER was observed in SPS rats.*P<0.01 vs. the control group.

In order to understand more about casapse-12 cellular location following activation, we performed western blot on cell fractions purified by differential centrifugation. *Figure* 6A shows a panel verifying the quality of fractionation.

In the SPS group, the expression of caspase-12 protein in the ER of the hippocampus exhibited thick band, increased at 1 day after SPS and peaked at 4 day after SPS. The expression of caspase-12 protein in cytoplasm and mitochondria of the hippocampal cells showed very tiny band and did not change between control group and SPS group (Fig. 6B).

### Caspase-12 mRNA Expression

**Figure 7:**
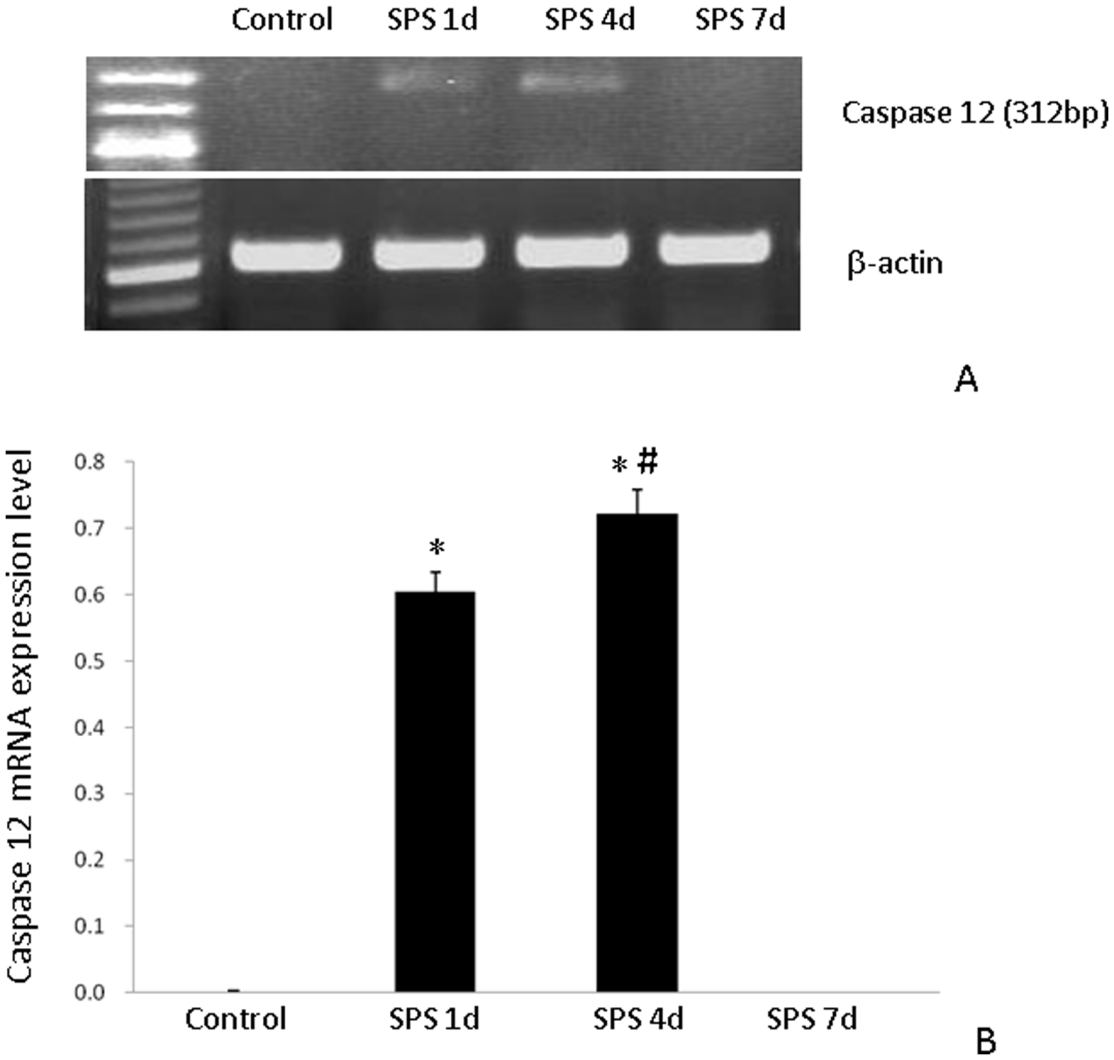
RT-PCR of Caspase-12 in the hippocampus of the SPS rats. Caspase-12 mRNA expression (A) and results from its quantitative analysis (B). Caspase-12 mRNA level in the hippocampus of rats subjected to SPS was higher than that in the hippocampus of control rats. *P<0.05 vs. the control group, #P<0.05 vs. the SPS 1 day group.

The mRNA expression of caspase-12 was not detected in the control group. At 1 day after SPS, caspase-12 mRNA expression level was significantly increased, peaked at 4 days after SPS, and then disappeared completely at 7 days after SPS (Fig. 7).

### Free Ca^2+^ Concentration in the Hippocampus

**Figure 8:**
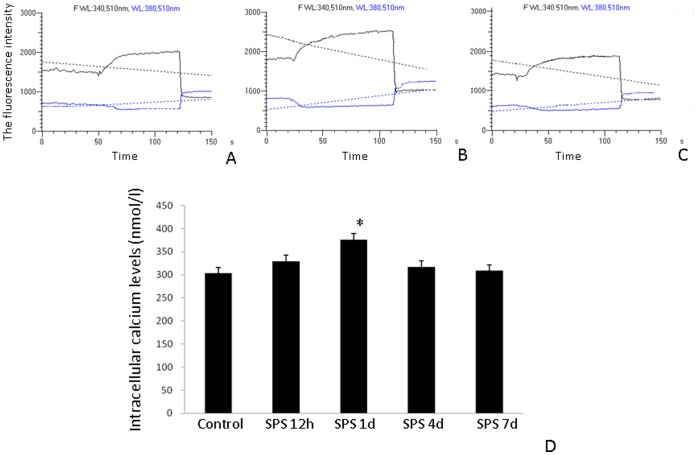
Change of intracellular calcium levels in the hippocampal cells of the SPS rats. Image of intracellular calcium levels in the control group (A), 1 day (B) and 4 days(C) after SPS and change in intracellular calcium (Ca^2+^) levels (nmol/l) from its quantitative analysis. *P<0.05 vs. the control group.

The intracellular free Ca^2+^ level in the hippocampus neurons was significantly higher in rats at 1 day after SPS than in control group and returned to normal levels 7 days after SPS (Fig. 8).

### mRNA Expression of CaM and CaMKIIα

**Figure 9:**
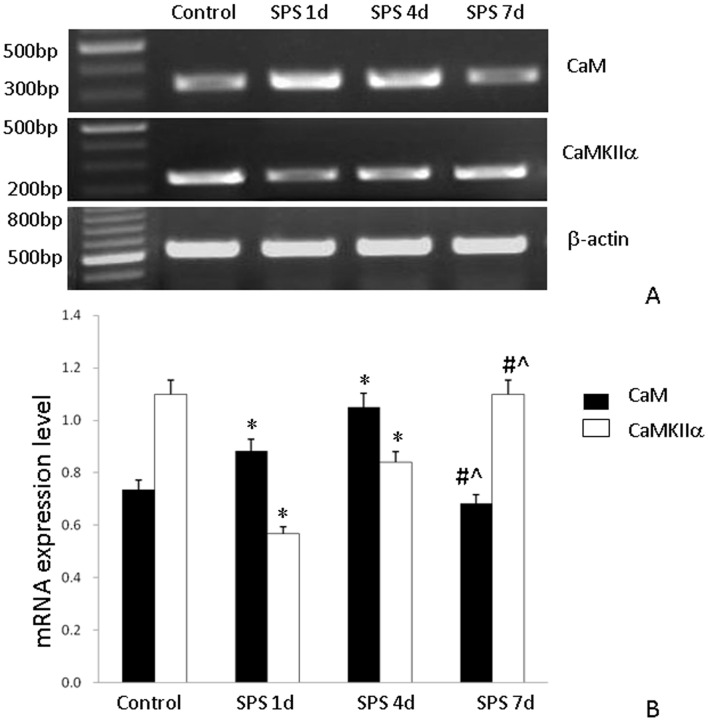
RT-PCR of CaM/CaMKIIa in the hippocampus of SPS rats. Calmodulin (CaM) and CaM kinase IIα (CaMKIIα) mRNA expression (A) and results from its quantitative analysis (B). Increased CaM and decreased CaMKIIα expression levels were observed in the hippocampus of rats subjected to SPS while CaM and CaMKIIα expression levels were unchanged in control rats. *P<0.05 vs. the control group, #P<0.05 vs. the SPS 1 day group, #P<0.05 vs. the SPS 4 days group.

The mRNA levels of CaM and CaMKIIα were normalized to the β-actin mRNA level. The mRNA expression level of CaM significantly increased at 1 day and 4 days after SPS but did not change in control rats, and then recovered to normal level at 7 days after SPS. In contrast, the mRNA expression level of CaMKIIα markedly decreased at 1 day and 4 days after SPS (Fig. 9A, 9B).

## Discussion

PTSD is a stress-related mental disorder caused by experience of a traumatic event, and presents with characteristic symptoms including re-experiencing symptoms (e.g., nightmares and flashbacks), hyperarousal symptoms (e.g., insomnia), numbing symptoms (e.g., restricted affect and anhedonia), avoidance symptoms (e.g., avoiding trauma-related stimuli), poor concentration and difficulty explicitly recalling aspects of the traumatic event. Magnetic resonance imaging studies showed decreased volume of the hippocampus in PTSD patients [30], suggesting a strong relationship between atrophy of the hippocampus and PTSD. Other studies from our team examined apoptosis in the hippocampus [9], amygdale [10] and cortex (unpublished data) of the single prolonged stress (SPS) rats. In this study, apoptotic cells were significantly increased in the hippocampus of the SPS rat as detected by TUNEL method. Consistent with this, apoptotic morphological changes were found in hippocampal neurons of SPS rats, including plasma membrane blebbing, cell shrinkage, chromatin condensation, and nuclear pyknosis. These observations indicate that SPS could induce neuronal apoptosis in hippocampus and that apoptosis may be one of the causes of hippocampal dysfunction.

Recently, it has been reported that endoplasmic reticulum also mediates apoptosis. The efficient functioning of the endoplasmic reticulum is essential for most cellular activities and survival. Many acute and chronic neurodegenerative disorders lead to accumulation of unfolded proteins and induce the unfolded protein response (UPR) [31]. The objective of UPR is to quickly reduce the requirement for ER protein processing and to eliminate the misfolded proteins as rapidly as possible. The protein degradation mechanism includes transporting the misfolded proteins out of the ER to the cytoplasm through a translocon. When the capacity of the ER to cope is saturated, UPR induces production of molecular chaperones (GRP78) and other components, and these proteins are required for the removal of the polypeptides that fail to fold properly [19], [32]. The overall result is improvement of folding and processing efficiency, and reduction in the flow of proteins into the ER compartment. If the adaptive capacity of the UPR to handle the problem is exceeded, the UPR response includes the induction of pro-apoptotic events including the up-regulation of a number of cell death genes including caspase-12 and their eventual activation. The three primary mammalian UPR ER-transmembrane proteins that control transcription, translation, and apoptosis are PERK, IRE1α, and ATF6. All three proteins are required for cell survival. Normally, GRP78 binds PERK, IRE1 and ATF6 and inhibits their activation in non-stressed cells [33]. When unfolded proteins in the ER lumen reach a critical level, GRP78 disassociates from PERK, IRE1αand ATF6 to bind the unfolded protein to prevent its aggregation [34], [35]. In this study, we showed that the mRNA and protein expression level of GRP78 increased at 1 day, peaked at 4 days, and decreased at 7 days after SPS. The increase in GRP78 expression at the early time points after SPS indicates GRP78 accumulation in the ER, which is the initial event to protect against SPS-induced apoptosis. The increase in GRP78 expression is beneficial because GRP78 binds unfolded proteins in order to eliminate denatured proteins and to re-establish cellular homeostasis. At 7 days after SPS, we observed a decrease in GRP78 expression and an increase in the number of TUNEL-positive cells, suggesting that SPS compromised ER function in the hippocampus and that ER-stress cannot be resolved through increased GRP78 expression, consequently leading to cell death. These data also confirm that an increase in GRP78 expression protects against ER stress-induced apoptosis. Recently, GRP78 has been intensively studied as the master regulator of ER stress. Prolonged ER stress leads to cell death and is linked to the pathogenesis of some neurodegenerative disorders including ischemia, Alzheimer’s and Parkinson’s diseases [36]. For example, following ischemic injury, the mRNA and protein expression of GRP78 in the hippocampus increased immediately after the injury, but then decreased at a later time [37]. However, it remains to be determined what the exact role of GRP78 in ameliorating PTSD. Our results suggest that understanding GRP78 function in the context of PTSD may reveal the mechanisms underlying PTSD pathogenesis.

Caspase-12 is localized to the ER and activated by ER stress, which includes disruption of calcium homeostasis and accumulation of unfolded proteins in the ER. It is not activated, however, by membrane- or mitochondrial-targeted apoptotic signals [17]. This suggests that caspase-12 is specific to ER-related pathways. Shibata and colleagues have found that concomitant to the temporal increase in GRP78 expression is the increase in the level of activated caspase-12 when the ER is stressed [38]. Interestingly, mice deficient in caspase-12 are resistant to ER stress-induced apoptosis [17]. In our study, caspase-12 mRNA expression level was unchanged in the control group. However, it increased in rats examined 1 day after SPS, remained at a high level at 4 days, but then disappeared at 7 days. It has been suggested that ER stress-dependent apoptotic cell death is caused through the activation of ER-specific caspase-12. The stimuli that induce ER stress also induce the recruitment of IRE1, which results in the disassociation of IRE1 from procaspase-12 and consequently, the oligomerization and activation of caspase-12. Thus the signaling pathway that initiates ER stress-induced apoptosis appears to depend on the ER-associated caspase-12 [17].

Several lines of evidence support the view that alterations in intracellular Ca^2+^ homeostasis are important in the apoptotic process [18], [39]. Some stress stimuli damage Ca^2+^ homeostasis. This can lead to either Ca^2+^ overload or deprivation, which can compromise ER function and protein synthesis, translation, and folding. The resulting abnormal proteins accumulate and then induce ER-stress. However, in some cell types, it has been reported that the depletion, rather than the overload, of ER Ca^2+^ stores is critical in apoptosis [40], [41]. Thus it is not entirely clear which components of the Ca^2+^ signaling cascade are important to trigger apoptosis. In our study, we observed calcium overload in the hippocampus 1 day after SPS, but the calcium level returned to normal level 4 days after SPS. These studies suggest that SPS compromises cellular Ca^2+^ homeostasis and that increased intracellular Ca^2+^ could induce ER stress. We also analyzed the level of ca^2+^/CaM, a ubiquitous Ca^2+^ sensor protein involved in almost all intracellular events. CaMKIIα is the molecular basis of learning and memory, but in the absence of bound Ca^2+^/CaM, CaMKIIα is in its inactive conformation. The influx of Ca^2+^ results in CaMKIIα activation. Ca^2+^/CaMKIIα is a major mediator of Ca^2+^ signaling and of particular importance in the brain, contributing significantly to the regulation of nerve functions, including learning and memory [42]. It has been speculated that CaMKIIα responds to a strong and/or repeated stimulus when the cellular Ca^2+^ concentration is relatively high. CaMKIIα is highly effective in synaptic plasticity and considered as one of the best candidates for a memory molecule [43]. Our result showed an increase in CaM level at 1 day after SPS, suggesting that the CaM content changed synchronously with changes in the Ca^2+^ concentration. This occurred as a result of the SPS increasing the intracellular free Ca^2+^ levels in the hippocampal cells which then induces the overexpression of CaM. The change in CaMKIIα from inactive to active decreased the CaMKIIα level in the hippocampus following SPS exposure.

Li from our team has found change in the expression level of cytochrome C in the SPS rats, suggesting SPS induced mitochondria-dependent apoptosis in rat hippocampus. It is not contradictory with our result. It is possible that not only mitochondrial pathway but also endoplasmic reticulum pathway participate in SPS-induced apoptosis. ER and mitochondria form close contacts at 20% of the mitochondrial surface [44]. The direct contact between ER and mitochondria are referred to as mitochondrial associated membranes (MAM) [45]. MAM have pivotal roles in numerous cellular functions including Ca^2+^ signaling, lipid transport, energy metabolism, and cell survival. The interaction between the two organelles is mediated by mitochondrial shaping proteins and key chaperones including calnexin, calreticulin, ERp44, ERp57, GRp75, and sigma-1 receptor. It has been reported prolonged ER stress up-regulated release of cytochrome C and induced change of the mitochondria membrane potential [46]. Anti-apoptotic protein Bcl-2/Bcl-XL inhibited apoptosis induced by ER stress [47].

### Conclusion

We found that single-prolonged stress induced apoptosis in the hippocampus of rats. Changes in the expression levels of GRP78, caspase-12 and Ca^2+^/CaM/CaMKIIα, indicate that endoplasmic reticulum pathway participates in SPS-induced apoptosis. In particular, the increase in the expression of GRP78, which protects against apoptosis, may provide important information for the pathogenesis and treatment of PTSD. However, more research is needed to better understand the molecular mechanisms underlying PTSD-induced apoptosis.
